# Supplementary material for: A Synthetic Cross-Species CD200R1 Agonist Suppresses Inflammatory Immune Responses In Vivo
Source: Mol Ther Nucleic Acids. 2018 Jul 3;12:350–8. doi: 10.1016/j.omtn.2018.05.023 (PMC6037911; doi:10.1016/j.omtn.2018.05.023)
Supplement: Document S1. Figures S1 and S2 and Table S1 [file mmc1.pdf]

OMTN, Volume *12*

Supplemental Information

A Synthetic Cross-Species CD200R1 Agonist Suppresses Inflammatory Immune Responses *In Vivo*

Aaron Prodeus, Amanda Sparkes, Nicholas W. Fischer, Marzena Cydzik, Eric Huang, Ismat Khatri, Ashley Young, Lindsay Woo, Chung Wai Chow, Reginald Gorczynski, and Jean Gariépy

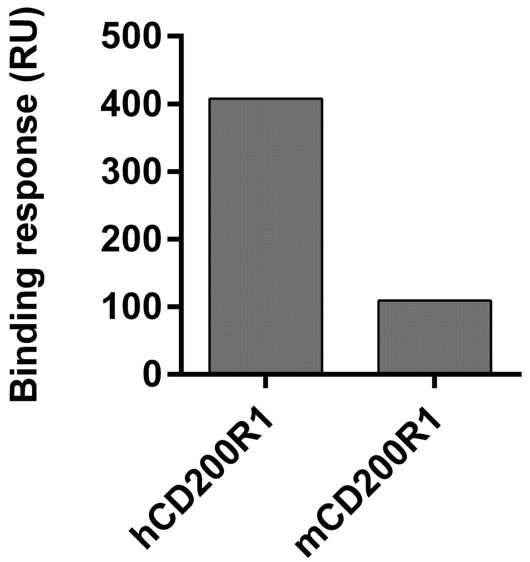

Supplemental Figure 1: CCS13 directly binds to both mouse and human CD200R1. Binding (RU; Response Units) of 5μM of either hCD200R1 or mCD200R1 to immobilized CCS13 as determined by SPR. Results shown are relative to an Fc control. CCS13 can bind both hCD200R1 and mCD200R1.

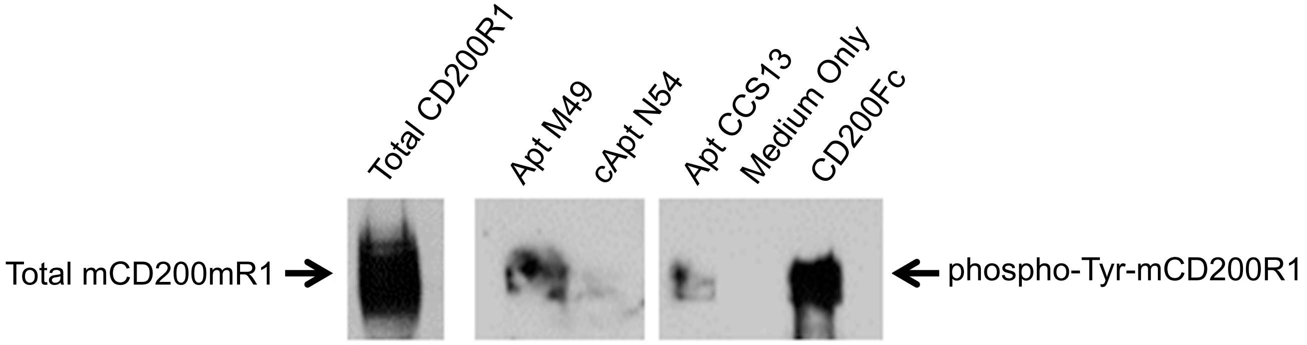

Supplemental Figure 2: CCS13 induces the phosphorylation of the murine CD200R1 cytoplasmic tail. HEK293 cells transfected to stably express mouse CD200R1 were incubated with a positive control, agonistic DNA aptamer M49 (1.5μM), a negative control aptamer cApt (3μM), or the CD200R1 cross species aptamer CCS13 (all 3μM). Phosphorylated and non-phosphorylated forms of CD200R1 were recovered from cell lysates by immunoprecipitation using an anti-murine CD200R1 (clone 2A10) monoclonal antibody. Total CD200R1 (loading control) was detected with a mAb to murine CD200R1 while a rabbit polyclonal antibody that recognizes the phosphorylated murine CD200R1 cytoplasmic tail was used to detect the phosphorylated form on western blots. CD200R1 expressing HEK293 cells were also treated with CD200Fc (3.3μM) or medium alone, serving as positive and negative controls respectively. Data are representative of two independent experiments.

Supplemental Table 1. TaqMan® Gene expression primers.

Gene	Assay ID
chemokine (C-C motif) ligand 11 (Eotaxin, CcL-11)	Mm00441238_m1
interleukin 13 (IL-13)	Mm00434204_m1
