# Supplementary material for: A Synthetic Cross-Species CD200R1 Agonist Suppresses Inflammatory Immune Responses In Vivo
Source: Mol Ther Nucleic Acids. 2018 Jul 3;12:350–8. doi: 10.1016/j.omtn.2018.05.023 (PMC6037911; doi:10.1016/j.omtn.2018.05.023)
Supplement: Document S2. Article plus Supplemental Information [file mmc2.pdf]

Molecular Therapy
Nucleic Acids
Original Article

AMERICAN SOCIETY of GENE & CELL THERAPY

A Synthetic Cross-Species CD200R1 Agonist Suppresses Inflammatory Immune Responses *In Vivo*

Aaron Prodeus,[1,2,6] Amanda Sparkes,[2,6] Nicholas W. Fischer,[1,2] Marzena Cydzik,[2] Eric Huang,[2] Ismat Khatri,[3] Ashley Young,[3] Lindsay Woo,[3] Chung Wai Chow,[3] Reginald Gorczynski,[4] and Jean Gariépy[1,2,5]

[1]Department of Medical Biophysics, University of Toronto, Toronto, ON M5G 1L7, Canada; [2]Physical Sciences, Sunnybrook Research Institute, Toronto, ON M4N 3M5, Canada; [3]Department of Medicine, University of Toronto, Toronto, ON M5G 2C4, Canada; [4]Departments of Surgery and Immunology, University Health Network, Toronto, ON M5G 2C4, Canada; [5]Department of Pharmaceutical Sciences, University of Toronto, Toronto, ON M5S 3M2, Canada

Functional aptamers displaying agonistic or antagonistic properties are showing great promise as modulators of immune responses. Here, we report the development of a polyethylene glycol-modified (PEGylated) DNA aptamer as a cross-species (murine and human) CD200R1 agonist that modulates inflammatory responses *in vivo*. Specifically, DNA aptamers were discovered by performing independent SELEX searches on recombinant murine and human CD200R1. Aptamer motifs identified by next generation sequencing (NGS) were subsequently compared, leading to the discovery of motifs common to both targets. The CD200R1 DNA aptamer CCS13 displayed the highest agonistic activity toward CD200R1 in terms of suppressing the induction of cytotoxic T-lymphocytes (CTLs) in both human and murine allogeneic-mixed lymphocyte cultures (allo-MLCs). A 20-kDa polyethylene glycol (PEG) chain was covalently attached to the 5′ end of this aptamer, and the resulting conjugate was shown to block inflammatory responses in murine models of skin graft rejection and house-dust-mite-induced allergic airway inflammation. Importantly, this agonistic aptamer does not suppress CTL induction in 5-day allo-MLCs with responder cells derived from CD200R1$^{-/-}$ mice, indicating that its mode of action is directly linked to CD200R1 activation. This study suggests that one can derive agonistic DNA aptamers that can be verified as immuno-modulators in murine models with outcomes potentially translatable to the treatment of human conditions.

INTRODUCTION

The immune system is a complex network of cells, immuno-regulatory molecules, and effector molecules aimed at: initiating the inflammatory immune response; coordinating cellular and humoral responses; and regulating mechanisms to regain homeostasis.[1,2] CD200R1 represents one such immuno-regulatory receptor on the surface of myeloid cells that, upon ligation to the cell-surface glycoprotein CD200, delivers immune inhibitory signals, leading to the suppression of mast cell and basophil degranulation[3] and the regulation of macrophage functions.[4] The physiological importance of this immuno-inhibitory pathway has been demonstrated in several inflammation models, including, but not limited to, experimental asthma,[5] transplantation,[6–9] inflammatory bowel disease,[10] neuropathic pain,[11] systemic lupus erythematosus,[12] arthritis,[13] and multiple sclerosis.[14,15] Consequently, the development of CD200R1-specific agonists for treating inflammatory conditions (e.g., autoimmunity, transplantation, airway hyperresponsiveness, and allergy) is of clinical importance. In this context, a CD200Fc fusion protein consisting of the extracellular domain of CD200 linked to an antibody Fc region was found to reduce disease severity in a murine model of experimental autoimmune encephalomyelitis (EAE) via the suppression of antigen-presenting cells and a reduction in pro-inflammatory cytokines.[14] CD200Fc was also shown to suppress neuro-inflammatory reactions associated with glial activation and neuropathic pain in rats,[11] to attenuate Toll-like receptor 4 (TLR4)-mediated renal inflammation in response to lipopolysaccharide (LPS) stimulation,[16] and to reduce the extent of collagen-induced arthritis in mice.[17] Alternatively, an agonistic CD200R1 antibody was shown to suppress pro-inflammatory cytokines in a model of experimental autoimmune uveoretinitis.[18]

Overall, the use of protein therapeutics as anti-inflammatory agents has had a major clinical impact,[19] although their high cost has led to the generation of biosimilars.[20] Furthermore, their immunogenicity[21] and their partial efficacy within patient populations and inflammatory diseases[22] have prompted searches for alternate classes of anti-inflammatory agents, including oligonucleotides.[23] Here, we derived DNA aptamers serving as CD200R1 agonists. This immune pathway targets mostly one arm of the immune system (myeloid

Received 10 September 2017; accepted 26 May 2018;
https://doi.org/10.1016/j.omtn.2018.05.023.

[6]These authors contributed equally to this work.

Correspondence: Jean Gariépy, PhD, Physical Sciences, Sunnybrook Research Institute, Toronto, ON M4N 3M5.
E-mail: gariepy@sri.utoronto.ca

This is an open access article under the CC BY license (http://creativecommons.org/licenses/by/4.0/).

Check for updates

A

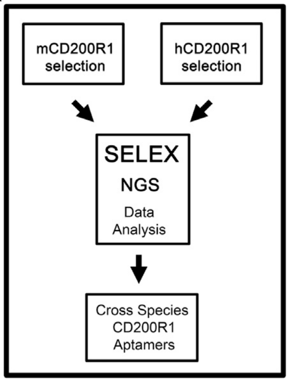

B

	% total reads in hCD200R1	% total reads in mCD200R1
CCS.1	2.76	0.40
CCS.2	1.85	0.22
CCS.4	1.40	0.07
CCS.5	0.52	0.57
CCS.8	0.15	0.98
CCS.10	0.12	0.04
CCS.11	0.04	0.21
CCS.13	0.05	0.01

Figure 1. Next-Generation Sequencing Performed on Separate Human and Murine CD200R1 SELEX Screens

(A) The selection strategy to identify to the presence of identical aptamer motifs. (B) The percentage of total sequence reads of each cross-reactive aptamer obtained from NGS sequence analysis. hCD200R1, human CD200R1; mCD200R1, murine CD200R1.

lineage) in contrast to present-day anti-cytokine-based antibody therapies (aimed at individual pro-inflammatory cytokines such as interleukin [IL]-1, tumor necrosis factor alpha [TNF-α], IL-6, IL-12, IL-17, IL-18, or IL-23).

Aptamers are small (<80 nt), single-stranded DNA or RNA oligonucleotides selected to bind target molecules with high affinity and specificity.[24–28] In particular, DNA aptamers can be rapidly and easily synthesized, as well as modified to increase their circulation half-life. They are inexpensive to produce, relative to protein therapeutics and, importantly, are non-immunogenic and lack toxicity.[24,26,28–34] In 2004, Macugen (pegaptanib) became the first aptamer (targeting vascular endothelial growth factor [VEGF]) to gain US Food and Drug Administration (FDA) approval for the treatment of age-related macular degeneration.[35] Several other aptamers are under clinical development for use as thrombolytic drugs, anti-obesity agents, autoantibody antagonists, and antiviral agents and for use in tumor diagnosis and therapeutics (as reviewed by Parashar[36]).

We have previously reported the successful *in vivo* use of agonistic CD200R1 aptamers to prolong major histocompatibility complex (MHC)-mismatched allograft survival in mice.[37] These aptamers, however, only recognized murine CD200R1 and, as such, had no potential to treat inflammatory diseases in humans. Herein DNA aptamer searches were performed on both human and murine CD200R1 which led to the development and characterization of a cross-species (murine and human) agonistic anti-CD200R1 DNA aptamer, termed CCS13. CCS13 exhibits *in vitro* immunosuppressive properties based on its ability to suppress cytotoxic T-lymphocyte (CTL) induction in both murine and human allogenic-mixed lymphocyte cultures (allo-MLCs). Furthermore, we demonstrate the *in vivo* therapeutic potential of a polyethylene glycol-modified (PEGylated) variant of CCS13 that prolonged the survival of murine skin allografts and suppressed the murine house-dust-mite allergic airway response.

RESULTS

Generation of Murine/Human Cross-Reactive CD200R1-Specific DNA Aptamers Displaying Agonistic Properties

Eight DNA aptamer sequences cross-reactive for murine and human CD200R1 were identified by analyzing sequence motifs arising after 15 SELEX (systemic evolution of ligands by exponential enrichment) enrichment rounds, performed separately on murine and human CD200R1 (Figures 1A and 1B). The aptamer sequences (75 nt long) were synthesized and screened for CD200R1 agonistic activity based on their ability to suppress CTL induction in murine and human 5-day allo-MLC assays. Aptamers termed CCS2, CCS5, CCS8, CCS10, and CCS13 displayed CD200R1 agonistic properties. CCS13, however, was the only DNA aptamer identified that was capable of agonizing both murine and human CD200R1 (Figure 2A). Consequently, CCS13 was chosen for further evaluation. Cross-reactivity of CCS13 was further confirmed by surface plasmon resonance (SPR) (Figure S1), whereby CCS13 was shown to directly bind to both murine and human CD200R1 with micromolar affinities. These findings are in range of the low micromolar affinity previously reported for the CD200:CD200R1 interaction[38,39] and our previously reported murine-specific anti-CD200R1 aptamers.[37]

PEGylated CCS13 Retains Agonistic Function

A 20-kDa linear polyethylene glycol (PEG) arm was coupled to the 5′ end of CCS13 (PEG-CCS13) to increase its circulatory half-life *in vivo*. To ensure that this modification did not alter its specificity for CD200R1 and its agonistic function, PEG-CCS13 and the PEGylated control aptamer (PEG-cApt) were re-evaluated in the allo-MLC assay. Here, the PEG-CCS13 aptamer was able to suppress CTL induction as compared to the control (PEG-cApt) (Figure 3A), confirming that PEGylation did not disrupt its immunosuppressive function. Importantly, increasing the concentration of PEG-CCS13 from 65 nM to 195 nM yielded similar immunosuppressive effects. Critically, the PEG-CCS13 agonistic function on CD200R1 signaling was confirmed, as it lacked the ability to suppress CTL lysis in lymphocytes isolated from CD200R1$^{-/-}$ mice (Figure 3B). To further characterize the mechanism by which CCS13 agonizes CD200R1, we examined whether it acted through the canonical CD200R1-mediated signal transduction pathway. Specifically, signaling is initiated upon binding of CD200 to CD200R1 on myeloid cells, leading to the phosphorylation of tyrosine 297 (murine CD200R1) on its C-terminal cytoplasmic tail.[40] As such, we monitored the ability of CCS13 to induce the phosphorylation of the CD200R1 cytoplasmic tail. Herein, HEK293 cells that were stably transfected to express murine CD200R1 were treated with CCS13 or a cApt medium alone or one of two positive controls (Apt M49, a murine-specific CD200R1 agonistic aptamer and CD200Fc[37]). In addition to the positive control, CCS13 led to the phosphorylation of the C-terminal tail of murine CD200R1, albeit to a lesser degree than the positive control (∼35% of CD200Fc) (Figure S2).

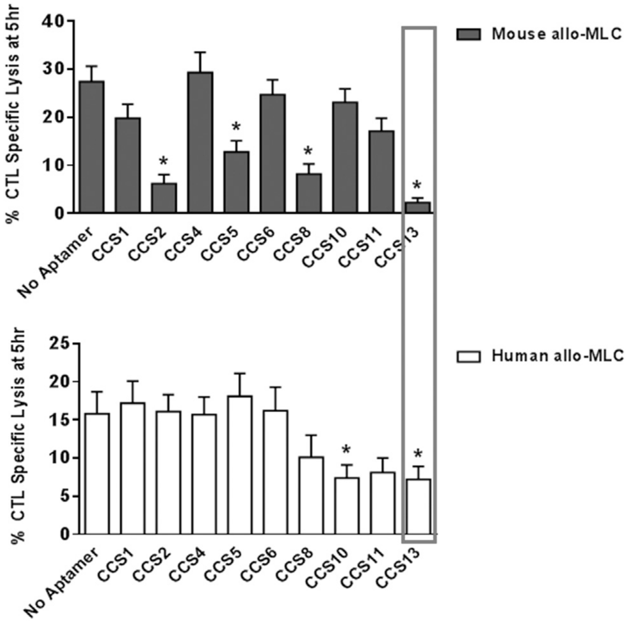

Figure 2. CCS13 Is a Cross-Species Agonist

The DNA aptamer CCS13 was the only common aptamer motif identified that was able to act as a CD200R1 agonist, suppressing allo-immune responses in both murine and human MLCs. Each bar represents the mean % value of CTL specific lysis ± SD. *p < 0.05, as compared to no aptamer.

PEGylated CD200R1 CCS13 Aptamer Prolongs Allogeneic Skin Graft Survival

The immunosuppressive effects of PEG-CCS13 *in vivo* were first evaluated in an acute murine skin-transplant rejection model. Here, BALB/c skin grafts were transplanted onto C57BL/6 mice, and the aptamers PEG-CCS13 and PEG-cApt or CD200Fc were subsequently administered as a series of intravenous (i.v.) injections every 3 days over a period of 15 days in combination with low-dose rapamycin given every 36 hr (Figure 4A). The administered dose of rapamycin has been previously shown not to affect graft survival.[37] Treatment with PEG-CCS13 protected the allograft from rejection as compared to the PBS or control aptamer (PEG-cApt) groups (Figure 4B). All treatments were discontinued after 15 days, as all skin grafts for the two control groups had been rejected. Importantly, the level of immunosuppression elicited by PEG-CCS13 in this transplantation model was comparable to that observed for CD200Fc. Additionally, splenocytes isolated from mice undergoing the graft rejection model were tested in the MLC assay. Here, splenocytes derived from mice previously treated with PEG-CCS13 or CD200Fc exhibited significant suppression of CTL induction as compared to the control groups (Figure 4C).

PEGylated CD200R1 CCS13 Aptamer Suppresses House-Dust-Mite-Induced Allergic Airway Inflammation

Given the success of PEG-CCS13 in prolonging allogeneic skin graft survival, a model that leads to an acute inflammatory response, we proceeded to assess whether this aptamer could be beneficial in a murine model of acute house-dust-mite (HDM)-induced allergic airway inflammation. This model is characterized by enhanced airway responsiveness to methacholine and increased airway inflammation. As expected, the total respiratory resistance (Rrs; mean ± SEM) increased with increasing doses of methacholine (MCh) in HDM-sensitized mice when compared to naive (saline control) mice (Figure 5A; *$p < 0.05$; n = 6–7 mice per group). A comparable reduction in MCh responsiveness of Rrs in the HDM mice was observed following the i.v. administration of either CD200Fc (positive control) or PEG-CCS13 when given after the establishment of the airway inflammation phenotype (Figure 5A; *$p < 0.05$; HDM + saline versus HDM + CD200Fc or HDM + PEG-CCS13, HDM subgroups; n = 6–7 mice per group). Reduction in the MCh responsiveness was most obvious at the maximal Rrs (Rrs_{max}) in the HDM mice following treatment with CD200Fc or PEG-CCS13 (Figure 5B; $p < 0.05$ for HDM versus saline; $p < 0.05$ for HDM + saline versus HDM + CD200Fc or HDM + PEG-CCS13, HDM subgroups; n = 6–7 mice per group). HDM sensitization increased pulmonary expression of IL-13, an important mediator of allergic inflammation, and eotaxin-1 (CCL-11), an eosinophil chemokine. Treatment with PEG-CCS13 following HDM sensitization significantly attenuated expression of both mediators (Figures 6A and 6B; $p < 0.05$ for saline versus HMD; $p < 0.05$ for HDM + saline versus HDM + PEG-CCS13, HDM subgroups; n = 6–7 per group). Together, these observations demonstrate that PEG-CCS13 can reverse HDM-induced airway hyper-responsiveness and allergic inflammation in an acute murine model of allergic airway inflammation.

DISCUSSION

Immune-mediated inflammatory disorders affect an estimated 5%–7% of the population in Western countries.[41–45] When left unmanaged, these disorders can also lead to the development of cancers.[41] Likewise, inflammatory immune responses remain a challenge for transplant patients, despite current immunosuppressive therapies.[46,47] Immune receptors and their ligands that potentiate or suppress inflammatory responses have emerged as attractive therapeutic targets. In this context, several studies to date have shown that the CD200:CD200R1 pathway exhibits an immunosuppressive effect on the inflammatory cellular response and contributes to the reduction of severity in animal models of inflammation and autoimmunity. For instance, soluble CD200 constructs, as well as an agonistic monoclonal antibody directed at CD200R1, have shown promise in preclinical experimental models of autoimmune encephalomyelitis[14] and autoimmune uveoretinitis[18] (in prolonging the survival of allografts[6]) and of collagen-induced arthritis.[17,48] It is reasonable, therefore, to consider the CD200:CD200R1 axis as a target for repression of inflammatory responses with potential application in allergy, autoimmunity, and transplantation. Herein, we developed a synthetic single-stranded DNA aptamer directed at both human and murine CD200R1 (mCD200R1) that acts as a CD200 mimic in dampening the pro-inflammatory responses associated with immune cells of the myeloid lineage. While several aptamers were identified as human and murine CD200R1 binders (Figure 1), only one aptamer termed CCS13 proved to be immunosuppressive in both murine and human allo-MLC assays (Figure 2A). This aptamer did not suppress CTL

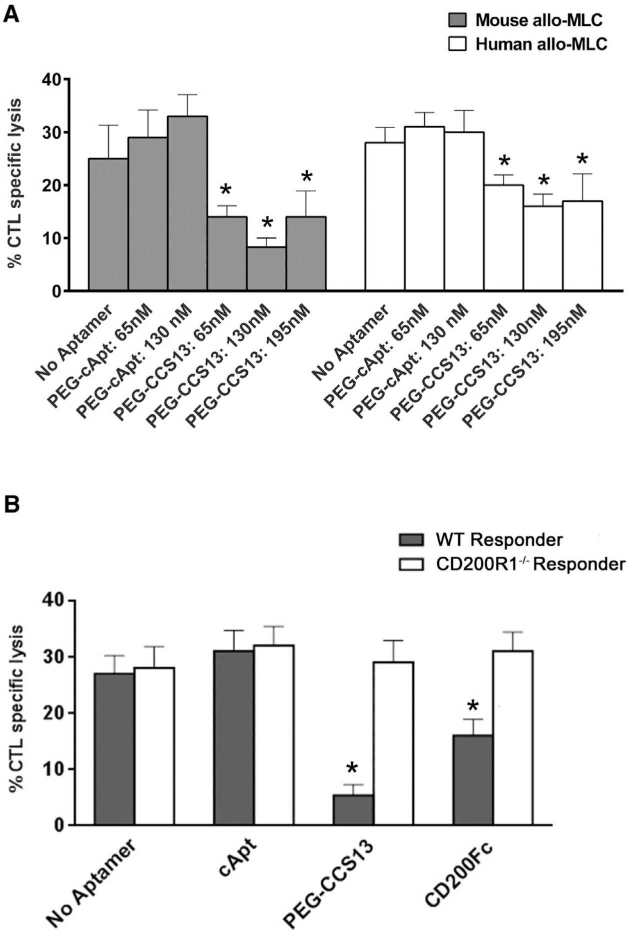

Figure 3. PEGylated Aptamer CCS13 Suppresses Allo-immune Responses through CD200R1

(A) Attachment of a 20-kDa linear polyethylene glycol appendage at the 3′ end of the DNA aptamer CCS13 does not abrogate its ability to block CTL activity in both murine and human allo-MLCs. (B) PEGylated CCS13 blocks the activity of murine lymphocytes recovered from WT C57BL/6 mice but not from CD200R1$^{-/-}$ mice. Each bar represents the mean % value of CTL specific lysis ± SD. *p < 0.05, as compared to no aptamer.

functions of lymphocytes derived from CD200R1$^{-/-}$ mice and rapidly induced the phosphorylation of mCD200R1 cytoplasmic tail, further suggesting that it is functioning as an agonist. Derivation of this CD200R1 cross-species DNA aptamer allows us to use *in vivo* pre-clinical murine models of inflammatory diseases as "proof of principle" for a therapeutically relevant compound that could later be translated to human diseases.

As in the case of small protein therapeutics such as antibody fragments, single chain variable fragments (scFvs), and soluble protein ligands, DNA aptamers are rapidly cleared from circulation through the renal system.[24] Consequently, we coupled a linear PEG arm to the 5′ end of CCS13 prior to evaluating this aptamer in *in vivo* murine models of inflammation. Modification with PEG has previously been proven to effectively improve the pharmacokinetic properties of DNA aptamers *in vivo*.[24,49,50] As projected, the PEGylated form of CCS13 (PEG-CCS13) retained its immunosuppressive function in both human and murine allo-MLC assays in a CD200R1-specific manner (Figures 3A and 3B, respectively). We subsequently assessed its capacity to suppress inflammatory responses in two distinct murine models of inflammation. In the first instance, PEG-CCS13 was able to prolong the survival of murine skin allografts (Figure 4). Here, the treatment of mice with PEG-CCS13 or CD200Fc was halted 15 days post-transplantation; the point at which all mice who had received PBS or an irrelevant PEGylated aptamer (PEG-cApt) had rejected their skin grafts. While survival was significantly prolonged in the PEG-CCS13 or CD200Fc and corresponded to a reduction in CTL response, all mice eventually rejected their grafts 6–8 days later—a possible reflection of the circulation half-lives of PEG-CCS13 and CD200Fc. Gorczynski and his colleagues[9] demonstrated that the continued CD200R1 stimulation using a doxycycline-inducible CD200 transgenic mouse line can induce full allograft acceptance with no need for further expression. This finding suggests that, by extending the treatment with PEG-CCS13, a greater proportion of mice may demonstrate a long-term acceptance of the graft. As a second model of inflammatory disease, we monitored the impact of PEG-CCS13 in the HDM-induced allergic airway inflammation model. This pre-clinical murine model has been used in the past to assess the therapeutic value of new drugs aimed at treating patients with asthma. The incidence of asthma, despite the availability of anti-inflammatory and bronchodilator therapies, is on the rise worldwide with >300 million people affected.[51] As was expected for this model,[52–54] airway hyper-responsiveness to MCh increased significantly in HDM-treated mice when compared to naive saline control mice (Figure 5). Remarkably, a single i.v. dose of PEG-CCS13, given after the establishment of airway inflammation, was capable of significantly reducing enhanced respiratory resistance to MCh to similar levels as treatment with CD200Fc. Additionally, in the HDM-sensitized mice, PEG-CCS13 reduced gene expression of IL-13 and eotaxin-1 (CCL-11), two important regulators of the allergic immune response (Figure 6).

Collectively, the data presented here demonstrate the ability of a PEGylated DNA aptamer to successfully agonize CD200R1 and exhibit immunomodulatory properties in two different models of inflammation. Our study represents the first report of a cross-species (murine and human) CD200R1 agonistic aptamer and suggests that PEG-CCS13 may, potentially, represent an alternative treatment for human inflammatory diseases. Future work will evaluate this aptamer in blocking both acute and chronic inflammation in other disease models of clinical importance, such as autoimmunity.

MATERIALS AND METHODS

Mice

Wild-type (WT) C57BL/6 and WT BALB/c mice were purchased from Jackson Laboratory (Bar Harbor, ME, USA). CD200R1$^{-/-}$ mice were generated as previously described[55] and bred in an accredited facility at the University Health Network.

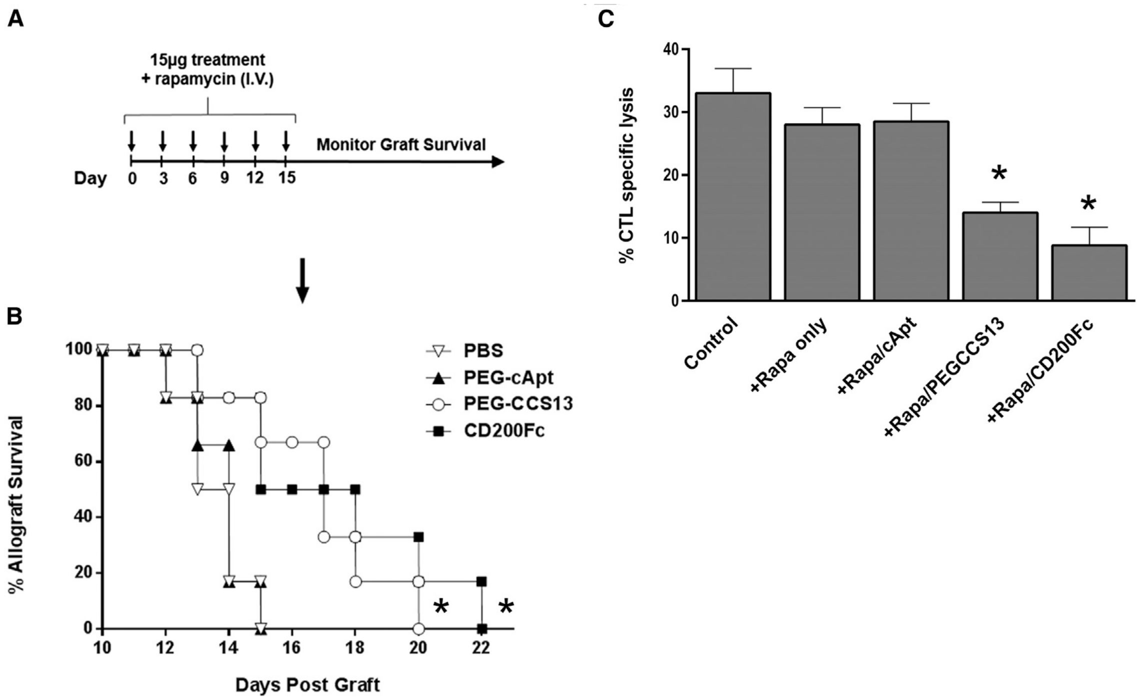

Figure 4. PEGylated Aptamer CCS13 Prolongs the Survival of Transplanted Murine Skin Grafts

(A) Experimental outline of *in vivo* experiment. C57BL/6 mice (n = 6) received BALB/c skin allografts on day 0 and were treated every 3 days over a period of 15 days with PBS or with 650 pmol PEG-cApt (negative control), 650 pmol PEG-CCS13, or 325 pmol CD200Fc in combination with low-dose rapamycin (0.5 mg/kg, 36 hr, i.p.). (B) Treatment with PEG-CCS13 (open circles) or CD200Fc (positive control; closed squares) significantly extended graft survival (*$p < 0.05$, Mann-Whitney U test) relative to the negative controls (closed triangles). Inverted open triangles represent PBS. Arrow represents the time of the last injection. Data shown are representative of two independent experiments. (C) CTL response is suppressed when using splenocytes isolated from mice previously treated with CCS13 or CD200Fc, as per the model indicated in (A). Each bar represents the mean % value of CTL specific lysis ± SD. *$p < 0.05$ as compared to the control; Student's t-test.

Aptamer Selection

The derivation of single-stranded DNA (ssDNA) aptamers recognizing murine and human CD200R1 followed a previously described SELEX protocol.[37,56,57] The synthetic oligonucleotide library consisted of a 25-nt-long random region flanked by 25-base-long 5′ and 3′ primer regions (5′-GACGATAGCGGT GACGGCACAGACGNNNNNNNNNNNNNNNNNNNNNNNNN NCGTATGCCGCTTCCGTCCGTCGCTC-3′). The primer sequences (forward: 5′-GACGATAGCGG TGACGGCACAGACG-3′ and reverse: 5′-GAGCGACGGAC GGAAGCGG CATACG-3′) were used for the PCR-based amplification of bound and recovered DNA oligonucleotides. The library (4 nmol; theoretical diversity: 425 or ~1.1 × 10^{15} sequences) was pre-adsorbed onto MagneHis Ni-Particles (Promega, Fitchburg, WI, USA) at 37°C for 1 hr to remove sequences that bound to the solid support itself. The remaining sub-library was then incubated for 1 hr at 37°C with either a recombinant His-tagged murine (10 µg) or human (10 µg) CD200R1 protein bound to a 1-mL suspension of MagneHis Ni-Particles (PBS; pH 7.4). The beads were then washed with PBS for 5 min to remove unbound and weakly bound sequences. Protein-aptamer complexes were eluted with 500 mM imidazole prepared in PBS. The ssDNA pools enriched for murine or human CD200R1 ligands were separately amplified for the next cycle by asymmetric PCR (10:1 forward:reverse primer ratio). Fifteen rounds of selection were performed, followed by NGS and motif identification, as described elsewhere.[32] Sequences that ranked within the top 50 in each murine or human selection were manually cross-compared with that of the opposite species to identify aptamers that shared an identical variable region.

SPR

CCS13 aptamer specificity to mCD200R1 and human CD200R1 (hCD200R1) was evaluated by SPR using the BIAcore T200 (GE Healthcare). A CM5 sensor chip (GE Healthcare) was first derivatized with a synthetic, 20-base-long poly(A) oligonucleotide carrying a 3′ amino group by amine coupling. CCS13 harboring a 20-base-long poly(dT) tail at its 5′ end was hybridized to the poly(A)-coated sensor chip to a level of 50–100 response units (RU). The immobilized aptamer and a reference flow cell, containing just the poly(A) oligonucleotide, were then exposed to injections (120 s, 30 µL/min) of mCD200R1 (5 µM), hCD200R1(5 µM), and Enbrel (5 µM) (Fc control) dissolved in HEPES-buffered saline (HBS)-P running buffer (10 mmol/L HEPES [pH 7.4], 150 mmol/L NaCl, 0.05% v/v Tween 20). Flow cells were regenerated by a 60-s pulse with 50 mM NaOH:1 M NaCl followed by a 5-min stabilization period.

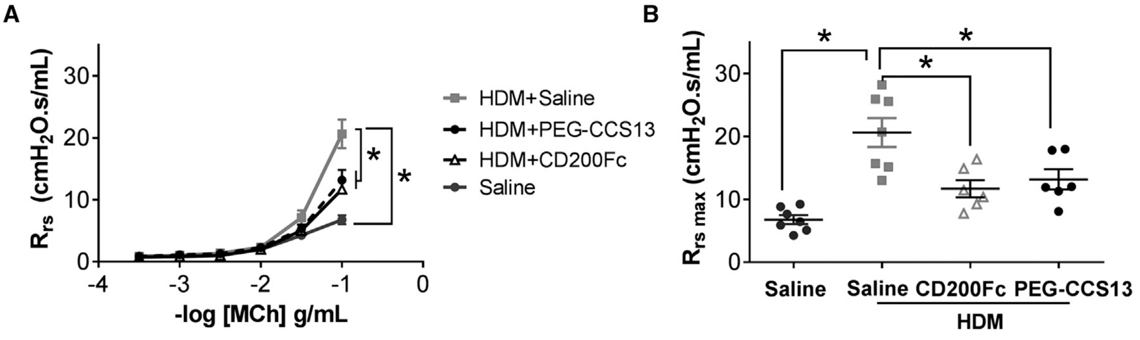

Figure 5. PEGylated Aptamer PEG-CCS13 Suppresses HDM-Induced Allergic Airway Response in BALB/c Mice

The methacholine (MCh) responsiveness of the total respiratory system resistance (R_{rs}) of BALB/c mice that had undergone a 2-week acute protocol of HDM-induced allergic airways inflammation is indicated and compared following treatment with a single i.v. dose of CD200Fc, PEG-CCS13, or saline. (A) HDM mice exhibited a dose-responsive increase in R_{rs} in response to increasing doses of inhaled MCh when compared to naive saline control mice (*$p < 0.05$; n = 6–7 mice per group). Intravenous treatment of the HDM mice with either CD200Fc or PEG-CCS13 significantly abrogated the MCh-induced increase in R_{rs}, as compared to non-treated HDM mice (*$p < 0.05$; n = 6–7 mice per group). (B) Reduction in the MCh responsiveness was most obvious at the maximal R_{rs} value (R_{rs} max) following treatment with CD200Fc and PEG-CCS13. Each data point represents a mean R_{rs} value ± SD. *$p < 0.05$; n = 6–7 mice per group.

Results were reported as response units after reference subtraction of RUs from the Fc control protein.

Detection of CD200R1 Phosphorylation

Phosphorylation of the cytoplasmic tail of murine CD200R1 by PEG-CCS13, and by PEG-cApt, was monitored using a rabbit polyclonal antibody that recognizes the phosphorylated form of CD200R1.[58] Serum-starved HEK293 cells stably expressing murine CD200R1 were maintained in OptiMEM (Life Technologies) medium for 3 hr and subsequently incubated for 30 min in the same medium containing either 1.5 μM PEG-M49 (positive control), 3 μM PEG-CCS13, 3 μM PEG-cApt (negative control), or 3.3 μM CD200Fc (positive control). Cells were washed with PBS and lysed in RIPA buffer (150 mmol/L NaCl, 1.0% Triton X-100, 0.5% sodium deoxycholate, 0.1% SDS, and 50 mmol/L Tris [pH 8.0]) containing 50 mmol/L NaF, 1 mmol/L Na_3VO_4, and protease inhibitors. Phosphorylated and non-phosphorylated forms of CD200R1 were recovered by immunoprecipitation using an anti-murine CD200R1 (clone 2A10) monoclonal antibody (overnight at 4°C) and protein G agarose beads (Pierce, Rockford, IL, USA). The total CD200R1 was detected from RIPA cell lysate by western blot using an anti-murine CD200R1 (clone 2A10) (1:1,000 dilution) and anti-rabbit horseradish peroxidase (1:15,000 dilution). The phosphorylated form of CD200R1 was detected by western blot using the rabbit polyclonal antibody specific to phosphorylated murine CD200R1 cytoplasmic tail (1:1,000 dilution) and anti-rabbit horseradish peroxidase (1:15,000 dilution). Densitometry analysis was performed using ImageJ 1.51k software.

PEGylation of DNA Aptamers

PEGylation of DNA aptamers was performed to increase their half-life in circulation. An amino group was incorporated during synthesis at the 5′ end of aptamer CCS13 and the cApt. A 20-kDa linear PEG chain was then covalently attached to each DNA aptamer (25 μM; dissolved in 100 mM $NaHCO_3$:CH_3CN [1:1, pH 8.5]) using a 100-molar excess of methoxy-PEG-succinimidyl glutarate ester (Creative PEGWorks, Winston-Salem, NC, USA) added dropwise over a period of 5 hr to each solution of amino-modified aptamers. The PEGylated aptamers were recovered by ultrafiltration and further purified by size exclusion chromatography using a Superdex 75 10/300 column (GE Healthcare) equilibrated in 100 mM NH_4CO_3. The resulting PEGylated aptamer conjugates were then lyophilized and stored at −80°C.

Murine and Human allo-MLCs

DNA aptamers found in both human and murine CD200R1 NGS datasets were synthesized to assess their ability to act as CD200R1 agonists. Specifically, eight cross-species CD200R1 DNA aptamers (Figure 1B) were evaluated for their ability to suppress the activation of CTLs in 5-day murine or human allo-MLCs. Experimentally, 2.5×10^5 C57BL/6 WT or CD200R1$^{-/-}$ responder splenocytes were incubated with an equal number of irradiated BALB/c stimulator cells in the presence or absence of synthetic cross-species aptamers for 5 days. Alternatively, 2.5×10^5 responder primary blood leukocytes (PBLs) and mitomycin-C-treated stimulator PBLs, obtained from individual human donors, were mixed in equal ratios. Percentages of CTL-specific lysis were determined by measuring the release of ^{51}Cr from loaded P815 mastocytoma target cells over a period of 5 hr at a 25:1 effector-to-target ratio.

Allogeneic Skin Graft Transplantation

The potency of PEG-CCS13 as an anti-inflammatory agent was assessed in terms of prolonging the survival of allogeneic murine skin grafts. Briefly, skin allografts from 8- to 12-week-old BALB/c mice were implanted onto C57BL/6 mice (n = 6). The recipient mice subsequently received 6 tail vein injections of either PEG-CCS13 (650 pmol), PEG-cApt (650 pmol), or CD200Fc (325 pmol) dissolved in 0.2 mL PBS (pH 7.4) once every 3 days for 15 days, given in combination with low-dose rapamycin (0.5 mg/kg, intraperitoneally [i.p.], every 36 hr). Splenocytes from treated mice were used in the MLC assay as described earlier.

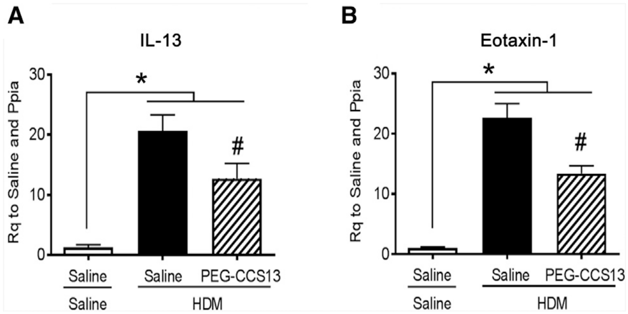

Figure 6. Treatment with PEG-CCS13 Attenuates Inflammatory Gene Expression in an Acute Model of Allergic Airway Inflammation

Pulmonary gene expression analysis (Rq, relative quantification) by qPCR revealed significantly increased expression of the allergic immune mediators IL-13 (A) and Eotaxin-1 (CCL-11) (B) in lung tissue of HDM-sensitized mice; treatment of HDM-mice with PEG-CCS13 reduced expression of both mediators compared to control treatment with saline. Each bar represents the mean gene expression value ± SD. *$p < 0.05$, HDM + saline versus saline control mice; $^{\#}p < 0.05$, PEG-CCS13 versus saline and HDM + saline subgroups; n = 6–7 mice per group.

HDM-Induced Allergic Airway Response

Following their acute exposure to the HDM (*Dermatophagoides pteronyssinus)* allergen (100 μg/35 μL saline via intranasal instillation daily on days 1–5 followed by a single dose at day 12), BALB/c mice (8–12 weeks old) were given a single i.v. dose of either CD200Fc (325 pmol), PEG-CCS13 (650 pmol), or saline (vehicle control). In addition, naive mice received 35 μL saline in the same dosing regimen to serve as a control group. Twenty-four hours after the i.v. treatment, baseline total respiratory system resistance (Rrs) in mice was measured using the flexiVent system, followed by evaluation of MCh responsiveness.[53,59] Right lungs of mice were extracted and stored in RNAlater (Life Technologies, Burlington, ON, Canada) at −20°C. To isolate RNA, lungs were homogenized, and RNA was extracted using the RNeasy Mini Kit (-QIAGEN, Toronto, ON, Canada) according to the manufacturer's instructions. In brief, ethanol is added to lung lysates allowing the selective binding of RNA to RNeasy membranes. Samples were placed in an RNeasy Mini Spin Column, and contaminants were washed away. Finally, RNA was eluted in RNase-free water. Reverse transcription was conducted using a QuantiTect Reverse Transcription Kit (QIAGEN, Toronto, ON, Canada) according to the manufacturer's instructions. Here, 1 μg isolated RNA was incubated for 2 min at 42°C with gDNA wipeout buffer. RNA was then incubated with the reverse-transcription master mix at 42°C for 15 min and then at 95°C for 3 min. cDNA was subsequently used in real-time qPCR using the CFX384 Touch Real-Time PCR Detection System (BioRad, Toronto, ON, Canada) with the following program: 50°C (2 min) and 40 cycles of 95°C (10 min), 95°C (15 s), and 60°C (1 min). Primer sets are presented in Table S1.

Statistical Analysis

The p values for survival analysis were determined using a Mann-Whitney U test, while other reported p values were calculated using a Student's t-test. Multiple comparisons were made with a 2-way ANOVA. All statistical analysis was done using GraphPad Prism 7.0.

SUPPLEMENTAL INFORMATION

Supplemental Information includes two figures and one table and can be found with this article online at https://doi.org/10.1016/j.omtn.2018.05.023.

AUTHOR CONTRIBUTIONS

Conceptualization, A.P. and J.G.; Methodology, C.W.C., J.G., and R.G.; Investigation, A.P., A.S., N.W.F., M.C., E.H., I.K., A.Y., and L.W.; Resources, J.G., R.G., and C.W.C.; Writing – Original Draft, A.S.; Writing – Review and Editing, J.G., A.P., and A.S.; Supervision, J.G., R.G., and C.W.C.

CONFLICTS OF INTEREST

The authors declare no competing financial interests.

ACKNOWLEDGMENTS

This work was financially supported by an operating grant from the Canadian Institutes of Health Research (CIHR MOP125862) to J.G. A.P. is the recipient of a Canadian Breast Cancer Foundation fellowship.

REFERENCES

1. Chaplin, D.D. (2010). Overview of the immune response. J. Allergy Clin. Immunol. *125* (2, Suppl 2), S3–S23.
2. Delves, P.J., and Roitt, I.M. (2000). The immune system. First of two parts. N. Engl. J. Med. *343*, 37–49.
3. Cherwinski, H.M., Murphy, C.A., Joyce, B.L., Bigler, M.E., Song, Y.S., Zurawski, S.M., Moshrefi, M.M., Gorman, D.M., Miller, K.L., Zhang, S., et al. (2005). The CD200 receptor is a novel and potent regulator of murine and human mast cell function. J. Immunol. *174*, 1348–1356.
4. Hoek, R.M., Ruuls, S.R., Murphy, C.A., Wright, G.J., Goddard, R., Zurawski, S.M., Blom, B., Homola, M.E., Streit, W.J., Brown, M.H., et al. (2000). Down-regulation of the macrophage lineage through interaction with OX2 (CD200). Science *290*, 1768–1771.
5. Lauzon-Joset, J.-F., Langlois, A., Lai, L.J., Santerre, K., Lee-Gosselin, A., Bossé, Y., Marsolais, D., and Bissonnette, E.Y. (2015). Lung CD200 receptor activation abrogates airway hyperresponsiveness in experimental asthma. Am. J. Respir. Cell Mol. Biol. *53*, 276–284.
6. Gorczynski, R.M., Hu, J., Chen, Z., Kai, Y., and Lei, J. (2002). A CD200FC immunoadhesin prolongs rat islet xenograft survival in mice. Transplantation *73*, 1948–1953.
7. Gorczynski, R.M., Cattral, M.S., Chen, Z., Hu, J., Lei, J., Min, W.P., Yu, G., and Ni, J. (1999). An immunoadhesin incorporating the molecule OX-2 is a potent immunosuppressant that prolongs allo- and xenograft survival. J. Immunol. *163*, 1654–1660.
8. Yu, K., Chen, Z., and Gorczynski, R. (2013). Effect of CD200 and CD200R1 expression within tissue grafts on increased graft survival in allogeneic recipients. Immunol. Lett. *149*, 1–8.
9. Gorczynski, R.M., Chen, Z., He, W., Khatri, I., Sun, Y., Yu, K., and Boudakov, I. (2009). Expression of a CD200 transgene is necessary for induction but not maintenance of tolerance to cardiac and skin allografts. J. Immunol. *183*, 1560–1568.
10. Elshal, M., Aldahlawi, A., Saadah, O., and McCoy, J. (2015). Reduced dendritic cells expressing CD200R1 in children with inflammatory bowel disease: correlation with Th17 and regulatory T cells. Int. J. Mol. Sci. *16*, 28998–29010.
11. Hernangómez, M., Klusáková, I., Joukal, M., Hradilová-Svíženská, I., Guaza, C., and Dubový, P. (2016). CD200R1 agonist attenuates glial activation, inflammatory

reactions, and hypersensitivity immediately after its intrathecal application in a rat neuropathic pain model. J. Neuroinflammation *13*, 43.

12. Li, Y., Zhao, L.D., Tong, L.S., Qian, S.N., Ren, Y., Zhang, L., Ding, X., Chen, Y., Wang, Y.X., Zhang, W., et al. (2012). Aberrant CD200/CD200R1 expression and function in systemic lupus erythematosus contributes to abnormal T-cell responsiveness and dendritic cell activity. Arthritis Res. Ther. *14*, R123.
13. Ren, Y., Leng, X.M., Yang, B., and Zhang, X. (2013). Aberrant CD200/CD200R1 expression contributes to painful synovium hyperplasia in a patient with primary hypertrophic osteoarthropathy. Rheumatol. Int. *33*, 2509–2512.
14. Liu, Y., Bando, Y., Vargas-Lowy, D., Elyaman, W., Khoury, S.J., Huang, T., Reif, K., and Chitnis, T. (2010). CD200R1 agonist attenuates mechanisms of chronic disease in a murine model of multiple sclerosis. J. Neurosci. *30*, 2025–2038.
15. Koning, N., Swaab, D.F., Hoek, R.M., and Huitinga, I. (2009). Distribution of the immune inhibitory molecules CD200 and CD200R in the normal central nervous system and multiple sclerosis lesions suggests neuron-glia and glia-glia interactions. J. Neuropathol. Exp. Neurol. *68*, 159–167.
16. Ding, Y., Yang, H., Xiang, W., He, X., Liao, W., and Yi, Z. (2015). CD200R1 agonist attenuates LPS-induced inflammatory response in human renal proximal tubular epithelial cells by regulating TLR4-MyD88-TAK1-mediated NF-κB and MAPK pathway. Biochem. Biophys. Res. Commun. *460*, 287–294.
17. Gorczynski, R.M., Chen, Z., Lee, L., Yu, K., and Hu, J. (2002). Anti-CD200R ameliorates collagen-induced arthritis in mice. Clin. Immunol. *104*, 256–264.
18. Copland, D.A., Calder, C.J., Raveney, B.J., Nicholson, L.B., Phillips, J., Cherwinski, H., Jenmalm, M., Sedgwick, J.D., and Dick, A.D. (2007). Monoclonal antibody-mediated CD200 receptor signaling suppresses macrophage activation and tissue damage in experimental autoimmune uveoretinitis. Am. J. Pathol. *171*, 580–588.
19. Strand, V., Kimberly, R., and Isaacs, J.D. (2007). Biologic therapies in rheumatology: lessons learned, future directions. Nat. Rev. Drug Discov. *6*, 75–92.
20. Jacobs, I., Petersel, D., Isakov, L., Lula, S., and Lea Sewell, K. (2016). Biosimilars for the treatment of chronic inflammatory diseases: a systematic review of published evidence. BioDrugs *30*, 525–570.
21. Strand, V., Balsa, A., Al-Saleh, J., Barile-Fabris, L., Horiuchi, T., Takeuchi, T., Lula, S., Hawes, C., Kola, B., and Marshall, L. (2017). Immunogenicity of biologics in chronic inflammatory diseases: a systematic review. BioDrugs *31*, 299–316.
22. Dinarello, C.A. (2010). Anti-inflammatory agents: present and future. Cell *140*, 935–950.
23. Paquet, J., Henrionnet, C., Pinzano, A., Vincourt, J.B., Gillet, P., Netter, P., Chary-Valckenaere, I., Loeuille, D., Pourel, J., and Grossin, L. (2011). Alternative for anti-TNF antibodies for arthritis treatment. Mol. Ther. *19*, 1887–1895.
24. Keefe, A.D., Pai, S., and Ellington, A. (2010). Aptamers as therapeutics. Nat. Rev. Drug Discov. *9*, 537–550.
25. Gilboa, E., McNamara, J., 2nd, and Pastor, F. (2013). Use of oligonucleotide aptamer ligands to modulate the function of immune receptors. Clin. Cancer Res. *19*, 1054–1062.
26. White, R.R., Sullenger, B.A., and Rusconi, C.P. (2000). Developing aptamers into therapeutics. J. Clin. Invest. *106*, 929–934.
27. Lakhin, A.V., Tarantul, V.Z., and Gening, L.V. (2013). Aptamers: problems, solutions and prospects. Acta Naturae *5*, 34–43.
28. Zhou, J., and Rossi, J. (2017). Aptamers as targeted therapeutics: current potential and challenges. Nat. Rev. Drug Discov. *16*, 440.
29. Kang, H., Ryu, S.H., Kahn, S.K., Oh, E.J., Park, J.K., and Kim, K.S. (2008). Characterization of PEGylated anti-VEGF aptamers using surface plasmon resonance. Macromol. Res. *16*, 182–184.
30. Cosmi, B. (2009). ARC-1779, a PEGylated aptamer antagonist of von Willebrand factor for potential use as an anticoagulant or antithrombotic agent. Curr. Opin. Mol. Ther. *11*, 322–328.
31. Conrad, R., Giver, L., Tian, Y., and Ellington, A.D. (1996). In vitro selection of nucleic acid aptamers that bind proteins. Methods Enzymol. *267*, 336–367.
32. Bouchard, P.R., Hutabarat, R.M., and Thompson, K.M. (2010). Discovery and development of therapeutic aptamers. Annu. Rev. Pharmacol. Toxicol. *50*, 237–257.
33. Healy, J.M., Lewis, S.D., Kurz, M., Boomer, R.M., Thompson, K.M., Wilson, C., and McCauley, T.G. (2004). Pharmacokinetics and biodistribution of novel aptamer compositions. Pharm. Res. *21*, 2234–2246.
34. Song, K.-M., Lee, S., and Ban, C. (2012). Aptamers and their biological applications. Sensors (Basel) *12*, 612–631.
35. Ng, E.W.M., Shima, D.T., Calias, P., Cunningham, E.T., Jr., Guyer, D.R., and Adamis, A.P. (2006). Pegaptanib, a targeted anti-VEGF aptamer for ocular vascular disease. Nat. Rev. Drug Discov. *5*, 123–132.
36. Parashar, A. (2016). Aptamers in therapeutics. J. Clin. Diagn. Res. *10*, BE01–BE06.
37. Prodeus, A., Cydzik, M., Abdul-Wahid, A., Huang, E., Khatri, I., Gorczynski, R., and Gariépy, J. (2014). Agonistic CD200R1 DNA aptamers are potent immunosuppressants that prolong allogeneic skin graft survival. Mol. Ther. Nucleic Acids *3*, e190.
38. Foster-Cuevas, M., Wright, G.J., Puklavec, M.J., Brown, M.H., and Barclay, A.N. (2004). Human herpesvirus 8 K14 protein mimics CD200 in down-regulating macrophage activation through CD200 receptor. J. Virol. *78*, 7667–7676.
39. Wright, G.J., Puklavec, M.J., Willis, A.C., Hoek, R.M., Sedgwick, J.D., Brown, M.H., and Barclay, A.N. (2000). Lymphoid/neuronal cell surface OX2 glycoprotein recognizes a novel receptor on macrophages implicated in the control of their function. Immunity *13*, 233–242.
40. Mihrshahi, R., Barclay, A.N., and Brown, M.H. (2009). Essential roles for Dok2 and RasGAP in CD200 receptor-mediated regulation of human myeloid cells. J. Immunol. *183*, 4879–4886.
41. Beyaert, R., Beaugerie, L., Van Assche, G., Brochez, L., Renauld, J.C., Viguier, M., Cocquyt, V., Jerusalem, G., Machiels, J.P., Prenen, H., et al. (2013). Cancer risk in immune-mediated inflammatory diseases (IMID). Mol. Cancer *12*, 98.
42. Davidson, A., and Diamond, B. (2001). Autoimmune diseases. N. Engl. J. Med. *345*, 340–350.
43. Jacobson, D.L., Gange, S.J., Rose, N.R., and Graham, N.M. (1997). Epidemiology and estimated population burden of selected autoimmune diseases in the United States. Clin. Immunol. Immunopathol. *84*, 223–243.
44. Peppercorn, M.A., and Cheifetz, A.S. (2017). Definition, epidemiology, and risk factors in inflammatory bowel disease. https://www.uptodate.com/contents/definition-epidemiology-and-risk-factors-in-inflammatory-bowel-disease.
45. Gelfand, J.M., Weinstein, R., Porter, S.B., Neimann, A.L., Berlin, J.A., and Margolis, D.J. (2005). Prevalence and treatment of psoriasis in the United Kingdom: a population-based study. Arch. Dermatol. *141*, 1537–1541.
46. Farrar, C.A., Kupiec-Weglinski, J.W., and Sacks, S.H. (2013). The innate immune system and transplantation. Cold Spring Harb. Perspect. Med. *3*, a015479.
47. (2017). Organ procurement and transplantation network. https://optn.transplant.hrsa.gov/.
48. Gorczynski, R.M., Chen, Z., Yu, K., and Hu, J. (2001). CD200 immunoadhesin suppresses collagen-induced arthritis in mice. Clin. Immunol. *101*, 328–334.
49. Haruta, K., Otaki, N., Nagamine, M., Kayo, T., Sasaki, A., Hiramoto, S., Takahashi, M., Hota, K., Sato, H., and Yamazaki, H. (2017). A novel PEGylation method for improving the pharmacokinetic properties of anti-interleukin-17A RNA aptamers. Nucleic Acid Ther. *27*, 36–44.
50. Da Pieve, C., Blackshaw, E., Missailidis, S., and Perkins, A.C. (2012). PEGylation and biodistribution of an anti-MUC1 aptamer in MCF-7 tumor-bearing mice. Bioconjug. Chem. *23*, 1377–1381.
51. Global strategy for asthma management and prevention 2006. (2006). http://www.who.int/respiratory/asthma/GINA_WR_2006_copyright[1].pdf.
52. Guo, W., Li, M.-R., Xiao, J.-J., and Huang, M. (2008). [Preparation and evaluation of mouse model of house dust mite-induced asthma]. Zhongguo Dang Dai Er Ke Za Zhi *10*, 647–650.
53. Salehi, S., Wang, X., Juvet, S., Scott, J.A., and Chow, C.-W. (2017). Syk regulates neutrophilic airway hyper-responsiveness in a chronic mouse model of allergic airways inflammation. PLoS ONE *12*, e0163614.
54. Piyadasa, H., Altieri, A., Basu, S., Schwartz, J., Halayko, A.J., and Mookherjee, N. (2016). Biosignature for airway inflammation in a house dust mite-challenged murine model of allergic asthma. Biol. Open *5*, 112–121.

55. Boudakov, I., Liu, J., Fan, N., Gulay, P., Wong, K., and Gorczynski, R.M. (2007). Mice lacking CD200R1 show absence of suppression of lipopolysaccharide-induced tumor necrosis factor-alpha and mixed leukocyte culture responses by CD200. Transplantation *84*, 251–257.

56. Tuerk, C., and Gold, L. (1990). Systematic evolution of ligands by exponential enrichment: RNA ligands to bacteriophage T4 DNA polymerase. Science *249*, 505–510.

57. Ellington, A.D., and Szostak, J.W. (1990). In vitro selection of RNA molecules that bind specific ligands. Nature *346*, 818–822.

58. Khatri, I., Boudakov, I., Lamptey, B., Taseva, A., Wong, K., Podnos, A., and Gorczynski, R. (2012). Structural and functional consequences of switching carboxy terminal domains in mouse CD200 receptors. Open J. Immunol. *2*, 168–186.

59. Penton, P.C., Wang, X., Amatullah, H., Cooper, J., Godri, K., North, M.L., Khanna, N., Scott, J.A., Chow, C.-W., et al. (2013). Spleen tyrosine kinase inhibition attenuates airway hyperresponsiveness and pollution-induced enhanced airway response in a chronic mouse model of asthma. J. Allergy Clin. Immunol. *131*, 512–520.e10.

OMTN, Volume *12*

Supplemental Information

A Synthetic Cross-Species CD200R1 Agonist Suppresses Inflammatory Immune Responses *In Vivo*

Aaron Prodeus, Amanda Sparkes, Nicholas W. Fischer, Marzena Cydzik, Eric Huang, Ismat Khatri, Ashley Young, Lindsay Woo, Chung Wai Chow, Reginald Gorczynski, and Jean Gariépy

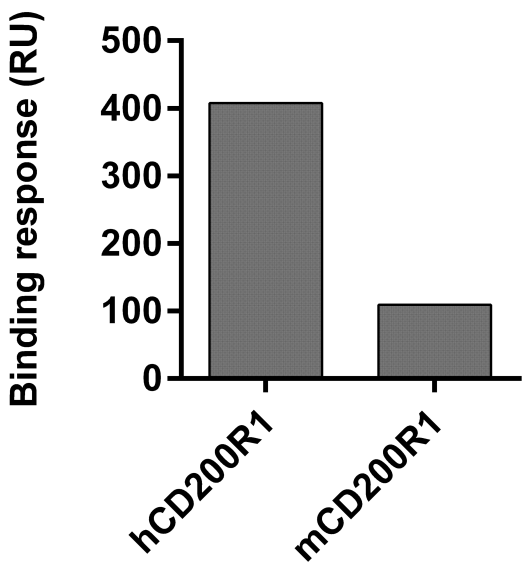

Supplemental Figure 1: CCS13 directly binds to both mouse and human CD200R1. Binding (RU; Response Units) of 5μM of either hCD200R1 or mCD200R1 to immobilized CCS13 as determined by SPR. Results shown are relative to an Fc control. CCS13 can bind both hCD200R1 and mCD200R1.

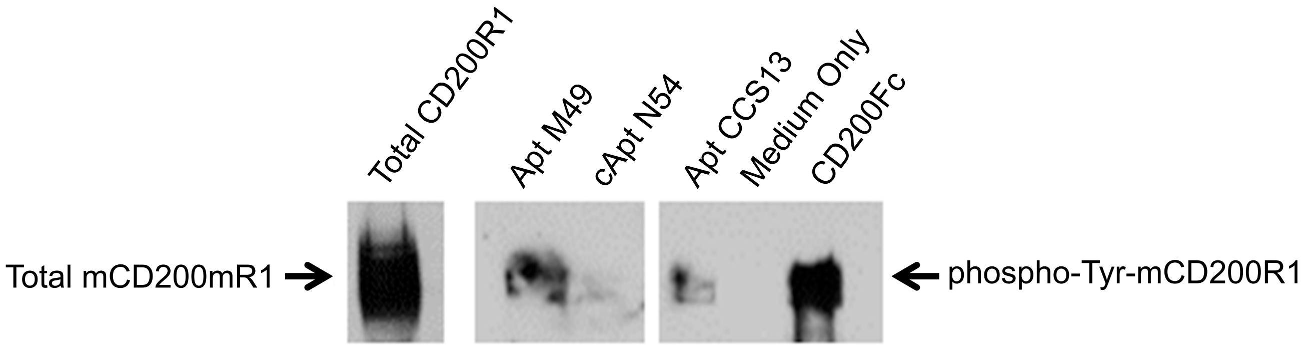

Supplemental Figure 2: CCS13 induces the phosphorylation of the murine CD200R1 cytoplasmic tail. HEK293 cells transfected to stably express mouse CD200R1 were incubated with a positive control, agonistic DNA aptamer M49 (1.5μM), a negative control aptamer cApt (3μM), or the CD200R1 cross species aptamer CCS13 (all 3μM). Phosphorylated and non-phosphorylated forms of CD200R1 were recovered from cell lysates by immunoprecipitation using an anti-murine CD200R1 (clone 2A10) monoclonal antibody. Total CD200R1 (loading control) was detected with a mAb to murine CD200R1 while a rabbit polyclonal antibody that recognizes the phosphorylated murine CD200R1 cytoplasmic tail was used to detect the phosphorylated form on western blots. CD200R1 expressing HEK293 cells were also treated with CD200Fc (3.3μM) or medium alone, serving as positive and negative controls respectively. Data are representative of two independent experiments.

Supplemental Table 1. TaqMan® Gene expression primers.

Gene	Assay ID
chemokine (C-C motif) ligand 11 (Eotaxin, CcL-11)	Mm00441238_m1
interleukin 13 (IL-13)	Mm00434204_m1
